# AhR Deletion Promotes Aberrant Morphogenesis and Synaptic Activity of Adult-Generated Granule Neurons and Impairs Hippocampus-Dependent Memory

**DOI:** 10.1523/ENEURO.0370-17.2018

**Published:** 2018-08-22

**Authors:** Juan de la Parra, María I. Cuartero, Alberto Pérez-Ruiz, Alicia García-Culebras, Ricardo Martín, José Sánchez-Prieto, Juan M. García-Segura, Ignacio Lizasoain, María A. Moro

**Affiliations:** 1 Unidad De Investigación Neurovascular, Dpto. Farmacología y Toxicología, Facultad De Medicina, and Instituto Universitario De Investigación En Neuroquímica (IUIN), Universidad Complutense De Madrid (UCM); Instituto De Investigación Hospital 12 De Octubre (i+12), Madrid, Spain; 2 Departamento De Bioquímica y Biología Molecular, Facultad De Veterinaria and IUIN, UCM, Madrid, Spain; 3 Departamento De Bioquímica y Biología Molecular, Facultad De Ciencias Químicas, and IUIN, UCM, Madrid, Spain

**Keywords:** Aryl hydrocarbon receptor, dendrite arborization, hippocampal memory, newborn granule cells

## Abstract

Newborn granule cells are continuously produced in the subgranular zone of dentate gyrus throughout life. Once these cells mature, they integrate into pre-existing circuits modulating hippocampus-dependent memory. Subsequently, mechanisms controlling generation and maturation of newborn cells are essential for proper hippocampal function. Therefore, we have studied the role of aryl hydrocarbon receptor (AhR), a ligand-activated bHLH-PAS transcription factor, in hippocampus-dependent memory and granule neuronal morphology and function using genetic loss-of-function approaches based on constitutive and inducible-nestin AhR^–/–^ mice. The results presented here show that the impaired hippocampus-dependent memory in AhR absence is not due to its effects on neurogenesis but to aberrant dendritic arborization and an increased spine density, albeit with a lower number of mature mushrooms spines in newborn granule cells, a finding that is associated with an immature electrophysiological phenotype. Together, our data strongly suggest that AhR plays a pivotal role in the regulation of hippocampal function, by controlling hippocampal granule neuron morphology and synaptic maturation.

## Visual Abstract

**Figure:**
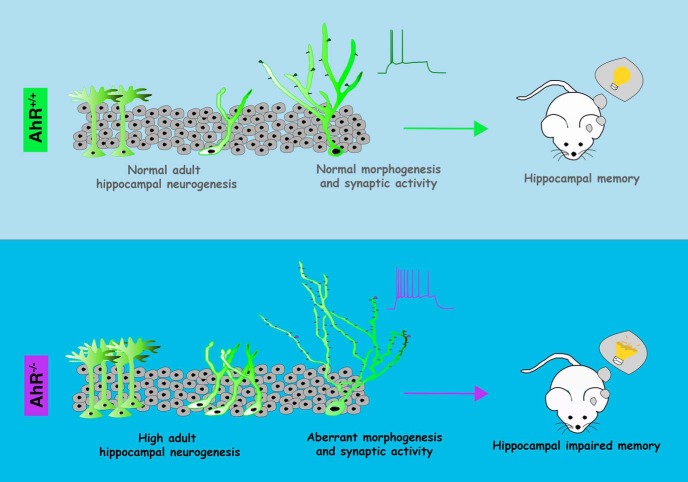

## Significance Statement

Hippocampus-dependent memory depends on the generation and maturation of dentate gyrus (DG) newborn granule cells. Aryl hydrocarbon receptor (AhR) is a ligand-activated bHLH-PAS transcription factor recently implicated in dendrite branching in the CNS. Because its role in the modulation of dendrite branching and plasticity of adult hippocampal newborn granule neurons and subsequent impact on hippocampus-dependent memory remains unknown, we have undertaken its study using genetic loss-of-function approaches in adult mice. Our study provides evidence indicating that AhR is a regulator of dendrite arborization and proper synaptic maturation of adult hippocampal newborn neurons and showing its critical role for learning and memory function. These findings point out AhR as a new potent druggable target for the treatment of several cognitive disorders.

## Introduction

In the adult mammal brain, the hippocampus is one of the main regions implicated in cognitive function. Throughout life, hippocampal newborn neurons migrate into the granule cell layer to become new dentate granule cells where they integrate synaptically into the pre-existing circuits providing potential substrates for new learning and memories (Altman and Das, 1965; Squire and Zola-Morgan, 1991; Kempermann et al., 1997; van Praag et al., 1999). The correct morphogenesis of these newborn neurons is a critical feature for the adequate function of the hippocampus. Although the aberrant integration of adult newborn granule neurons is able to disrupt cognitive function and is linked to several neurologic disorders (Zhou et al., 2013; Winkle et al., 2016), the molecular factors that drive the morphogenesis and maturation of these cells are still quite unknown.

The aryl hydrocarbon receptor (AhR), a ligand-activated transcription factor that belongs to the basic helix-loop-helix Per-ARNT-Sim (bHLH-PAS) superfamily, has been traditionally studied in association with toxic effects of environmental pollutants and xenobiotic compound metabolism and its role in the immune system (Fernandez-Salguero et al., 1995, 1996; Mandal, 2005; Murray et al., 2014; Mulero-Navarro and Fernandez-Salguero, 2016). However, recent studies across different species suggest other important biological roles of AhR in other systems, such as the CNS. In invertebrates, AhR homologs are implicated in dendrite branching in neurons: in *Drosophila*, the loss of function of the AhR homolog (*spineless*) promotes more complex dendritic arborization in sensory neurons (Crews and Brenman, 2006; Kim et al., 2006); in *C. elegans*, *ahr-1* mutant (AhR homolog) neurons also turn into a highly branched architecture (Smith et al., 2013). In mammals, AhR is expressed in the adult brain (Kimura and Tohyama, 2017), and its constitutive activation drastically reduces dendritic arborization and aberrant neuronal positioning in cortical pyramidal neurons and olfactory bulb interneurons (Kimura et al., 2016, 2017; Kimura and Tohyama, 2017). AhR mRNA is also expressed in the dentate gyrus (DG) granule cells of the adult hippocampus (Kimura and Tohyama, 2017). In this area, although it has been reported that AhR might modulate hippocampal neurogenesis (Latchney et al., 2013), its role in the modulation of dendrite branching and plasticity in adult hippocampal newborn granule neurons remains unknown. Therefore, we have studied the role of AhR in hippocampus-dependent memory and granule neuronal morphology and function using genetic loss-of-function approaches in adult mice. Our data demonstrate that the transcription factor AhR plays a crucial role in hippocampus-dependent function, by controlling dendritic arborization and dendritic spine growth in granule neurons.

## Materials and Methods

### Animals and tamoxifen treatment

Experiments were performed in male WT and AhR^–/–^ knockout mice (C57BL/6) at 4, 8, and 14 weeks of age, obtained from Taconic. Both WT and AhR^–/–^ mice were generated by crossing heterozygous AhR^±^ mice. AhR^f/f^ mice were acquired from The Jackson Laboratory and were maintained through homozygous breeding pairs. AhR icKO mice (tamoxifen-inducible AhR conditional knockout mice) were generated by crossing AhR^f/f^ mice (Walisser et al., 2005) with nestin-Cre^ERT2^ mice (Imayoshi et al., 2008) and then maintained through homozygous breeding pairs on a C57BL/6 background. In these transgenic mice (nestin-Cre^ERT2^/AhR^f/f^), tamoxifen treatment suppresses the expression of AhR in the neuroprogenitor cells present at the hippocampal subgranular zone (SGZ). The tamoxifen protocol used in this study was as described before (Cancino et al., 2013). Briefly, both AhR^f/f^ and AhR-icKO mice were administered tamoxifen intraperitoneally in two different rounds. The first round was performed at postnatal day 30 (p30) and the second at p60, each round consisting of a daily injection of tamoxifen (180 mg/kg) in sunflower oil for 5 consecutive days. Behavioral and histologic analyses were performed 3 weeks after the last tamoxifen administration. Mice had access to rodent chow and water *ad libitum* in a 12 h light/dark cycle room. This study was approved by the Animal Welfare Committee of the Universidad Complutense of Madrid, Spain.

### BrdU treatment

For quantification of the proportion of proliferating SGZ neural precursors, a total of 4 injections of the cell proliferation marker BrdU (5-bromo-2′-deoxyuridine; 100 mg/kg; Sigma-Aldrich) were administered intraperitoneally every 2 h to 4, 8, and 14-week-old control and AhR^–/–^ mice. 24 hours after the last administration, mice were sacrificed.

For quantification of the integrated adult newborn neurons (BrdU^+^/calbindin^+^ cells), 8-week-old WT and AhR^–/–^ mice were injected daily with BrdU (100 mg/kg) intraperitoneally for 5 consecutive days, and mice were sacrificed 28 days after the last administration.

### Histology

For histology and immunohistochemistry studies, mice were perfused transcardially with 0.1 m PBS followed by 4% paraformaldehyde (PFA) in 0.1 m PBS (pH 7.4). Brains were postfixed in PFA and transferred to 30% sucrose. For SVZ (from bregma +1.70 mm to bregma 0.02 mm) and dentate gyrus (DG; from bregma –1.46 mm to bregma –2.03 mm), coronal sections (30 µm) were cut using a microtome (Leica SM2000R) and stored in cryoprotective solution. Unless indicated otherwise, brain samples from AhR^–/–^ knockout and AhR icKO mice after tamoxifen treatment were analyzed at 2 and 3 months of age, respectively.

#### Immunohistochemistry

Immunofluorescence was performed on free-floating sections. Briefly, sections were first permeabilized and blocked in 0.25% Triton X-100 in PBS with 10% normal serum for 1 h and then incubated overnight at 4°C with the following primary antibodies in 0.25% Triton X-100 in PBS with 5% normal serum: goat anti-calbindin (neuronal marker; 1:500, Santa Cruz), goat anti-DCX (doublecortin; neuroblast marker; 1:250, Santa Cruz), rabbit anti-Ki67 (nuclear protein specifically expressed in cells undergoing active proliferation; 1:500, Abcam), chicken anti-GFAP (glial fibrillary acidic protein; astrocyte marker; 1:750, Thermo Scientific), mouse anti-nestin-PE (neural stem cell marker; 1:50, BD Biosciences), rabbit anti-AhR (1:200, Enzo Life Sciences), and chicken anti-GFP (1:700, Thermo Scientific). For BrdU staining, free-floating sections were pretreated with 2 N HCl for 30 min at 37°C and, after blocking in 0.25% Triton X-100 in PBS with 10% normal serum for 1 h, incubated overnight at 4°C with rat monoclonal anti-BrdU (1:200, Abcam) in 0.25% Triton X-100 in PBS with 5% normal serum. The secondary antibodies used were donkey Alexa-488 anti-goat (1:500, Invitrogen), donkey Cy3 anti-mouse (1:500, Vector Laboratories), goat anti-rat biotinylated (1:250, Vector Laboratories), streptavidin Alexa-488 conjugate (1:500, Thermo Scientific), goat Alexa-647 anti-chicken (1:500, Thermo Scientific), donkey Cy3 anti-rabbit (1:500, Thermo Scientific), and donkey Alexa-488 anti-chicken (1:500, Thermo Scientific) in 0.25% Triton X-100 in PBS with 5% normal serum. Controls performed in parallel without primary antibodies showed very low levels of nonspecific staining.

#### Image processing and quantitative analysis of immunostained sections

Image acquisition was performed with a laser-scanning confocal imaging system (Zeiss LSM710) and image analysis was accomplished with the ZEN2009 software (Zeiss). Image quantification was performed with ImageJ software (NIH) and Volocity software (Improvision). Ki67^+^, DCX^+^, nestin^+^, and BrdU^+^ cells were counted in confocal z-stack images. In all cases, quantification was performed using nonstereological methods. Specifically, every 5th section (30 µm, separated 150 µm apart) was selected for a total of 5 representative matched sections per hippocampus (from bregma –1.46 mm to bregma –2.06 mm) and SVZ (from bregma +1.70 mm to bregma 0.02 mm). Cells were counted manually in frames of 212.55 × 212.55 µm (1024 × 1024), and data were expressed as the number of cells per 1000 µm^2^. Because assessment of 5 sections may not reflect changes in hippocampal size and extent, quantification data should not be considered in terms of absolute numbers. Although stereological assessment would be more accurate, the fact that cell numbers in AhR^–/–^ mice ranged from being in excess to no differences with age suggests that the methods we have employed are sensitive to major changes that occur with development. Colocalization of calbindin^+^/BrdU^+^, nestin^+^/BrdU^+^, nestin^+^/GFAP^+^, and nestin^+^/GFAP^+^/BrdU^+^ was confirmed by orthogonal projection of z-stack files.

In p60 AhR^–/–^ and p90 AhR-icKO mice and their respective controls, apical dendrite length of DCX^+^ cells was assessed in 5 serial sections (30 µm) from dorsal hippocampus (from bregma –1.46 mm to bregma –2.06 mm). Apical dendrite (considered the segment between the soma and the first dendrite ramification) was manually traced in confocal z-stack images taken at 63×, and then dendritic length was measured by ImageJ. A total of 20–50 cells per animal from each group were quantified.

DCX^+^ dendritic staining was performed in confocal z-stack images taken at 40×. Briefly, rectangular regions of interest (ROIs) were generated around neuroblast dendritic arborization and somas. Total dendritic arborization (distribution pattern of neuroblast dendrites along granular and molecular layers of the DG) was also subdivided in 2 ROIs for differentiating proximal (GL) and distal (ML) DCX^+^ dendritic staining. Integrated density was quantified in each compartment after background subtraction. Total dendritic arborization/soma, GL/soma, and ML/soma ratios were calculated from these values. This method was internally normalized for immunostaining variability, since immunofluorescence values were always acquired in pairs of dendrites and adjacent somas.

### *Ex vivo* flow cytometry from SGZ- and SVZ-derived NPCs

To quantify the proportion of proliferating SGZ or SVZ neural precursors, mice were injected 4 times with 100 mg/Kg BrdU intraperitoneally every 2 h and sacrificed 24 h later. Brains were rapidly removed, and SVZ- and SGZ-derived NPCs of WT and AhR^–/–^ mice were dissected, placed in ice-cold PBS, and dissociated into a single-cell suspension. Cell suspensions were filtered on 40-µm nylon mesh strainers and centrifuged at 300 × *g* for 10 min at room temperature. Next, cells were fixed, permeabilized, and stained with anti-BrdU-APC and anti-Nestin-PE according to manufacturer´s instructions (BD Cytofix/Cytoperm Kit, BrdU Flow Kits BD Biosciences). Finally, cells were washed and resuspended in 300 µl FACS Flow (BD PharMingen); isotype controls (Miltenyi) were run in parallel. Whole suspensions were examined in a FACSCalibur flow cytometer using CellQuest software (BD PharMingen), and data were analyzed using FlowJo software (Tree Star),

### Golgi–Cox staining

The fresh brains from 8-week-old AhR^–/–^ and WT mice without perfusion were used for Golgi–Cox staining with FD Rapid GolgiStain Kit (FD Neuro Technologies) according to the user manual. Briefly, the brain was first placed in impregnation solution for 2 weeks followed by 2 days in 30% sucrose. Then, brains were cut into 100-µm coronal sections using a vibratome (Leica VT1000s) and stained. Neuronal reconstructions from each animal were randomly drawn at 40× magnification for the different analyses by using the Neurolucida neuron tracing system (Microbrightfield). Determination of total dendritic length of branches and Sholl analysis were performed by using Neuroexplorer software (Microbrightfield). Sholl analysis was conducted by counting the number of dendrites that crossed a series of concentric circles at 10-µm intervals from the cell soma. To calculate spine density of Golgi-stained neurons in the DG, a random 10-µm-long dendrite segment in the molecular layer was measured (100–200 µm from the soma) and total number of spines was traced. The spine density was determined by dividing the total number of spines by the 10-µm length of the dendritic segment.

### Retrovirus-mediated labeling and mophologic analysis of GFP^+^-newborn neurons

New neurons were labeled using a murine Moloney leukemia virus-based retroviral vector (CAG-GFP, a gift from Fred Gage, Salk Institute, La Jolla, CA; Zhao et al., 2006). Concentrated viral solutions were prepared by transfection of retroviral vectors into Gryphon Eco cells, followed by ultracentrifugation of viral supernatant and concentrated virus solution by ultraspeed centrifugation (average 3 × 10^7^ IU/ml). Mice (8 weeks old) were anesthetized with isoflurane and placed in a stereotaxic frame. 2 µl retrovirus was infused at a rate of 0.2 µl/min into the DG (–2 mm AP, –1.4 mm ML relative to bregma, and 2.4 mm DV from skull) with a 5-µl 32-gauge Hamilton syringe. After the infusion, we allowed 5 extra min to avoid the retrovirus flow during syringe releasing. 28 days after the viral infusion, mice from all groups were sacrificed for GFP morphologic experiments.

For morphometric analysis, 50-µm sections were used. A total of 10 sections of two series from each animal were used for immunohistochemical detection of GFP-labeled neurons. 8–10 randomly selected neurons from each group were reconstructed. Confocal 40× stacks of images were obtained, and z-projections were analyzed to determine total dendritic length and to perform Sholl analysis. All cells were traced using NeuronJ plugin for ImageJ software. Sholl analysis was performed to determine dendritic complexity using the plugin Sholl Analysis for ImageJ (Ferreira et al., 2014).

Primary apical dendrite length was manually traced from the soma until the first dendrite ramification point in randomly selected neurons and measured by ImageJ.

For spine analysis, images of GFP-labeled dendritic processes at the outer molecular layer were acquired at 0.5-µm intervals with a Zeiss LSM710 confocal microscope with a plan apochromatic 63× oil lens [numerical aperture (NA), 1.4] and a digital zoom of 3×. The lsm image files were subjected to two iterations of deconvolution with the AutoDeblur program (AutoQuant). The length of each dendritic segment was measured, and total number of spines in proximal (50–100 µm from the soma) and distal (100–200 µm from the soma) dendritic segments were manually counted. The spine density was determined by dividing the total number of spines by the length of the dendritic segment.

Mushroom spines in proximal (50–100 µm from the soma) and distal (100–200 µm from the soma) dendritic segments were identified when the estimated surface area (π × *D_major_* × *D_minor_*/4) was ≥0.4 µm^2^ (Zhao et al., 2006, 2014). For the quantification, 8–12 dendritic segments from each animal per group were used. Confocal imaging and data quantification were performed blinded to the experimental conditions.

### Electrophysiology experiments

AhR^–/–^ and WT mice (30 to 45 days old) were anaesthetized with isoflurane (1.5%–2% in a mixture of 80% synthetic air/20% oxygen) and decapitated. The brain was quickly removed and placed in ice-cold artificial CSF (ACSF) containing (in mM): NaCl 124, KCl 2.69, KH_2_PO_4_ 1.25, MgSO_4_ 2, NaHCO_3_ 26, CaCl_2_ 2, ascorbic acid 0.4, and glucose 10, continuously bubbled with carbogen (95% O_2_ and 5% CO_2_; pH 7.3). Sagittal hippocampal slices (325 μm thick) were obtained using a Leica VT 1200S vibratome and incubated (≥1 h) in a holding chamber at room temperature (21–24°C) in ACSF. Slices were transferred to an immersion recording chamber and superfused at 1 mL/min with gassed ACSF including 50 µM picrotoxin to block GABA_A_ receptors. Experiments were performed at 25°C by using a temperature controller (Warner Instruments). Granule cells from dentate gyrus were visualized under a 40× water immersion objective and a Nomarski condenser combined with infrared microscopy using differential interface contrast (DIC) in an Eclipse FN1 Nikon microscope. Whole-cell electrophysiological recordings from granule cells were performed using patch pipettes (3–4 MΩ resistance) pulled from thick-walled borosilicate glass (1.5 mm outer diameter and 1.1 mm inner diameter) on a P-97 puller (Sutter-Instrument) and filled with the internal solution containing (in mM): K-Gluconate 135, KCl 10, HEPES 10, MgCl_2_ 1, ATP-Na_2_ 2 (pH 7.3 adjusted with KOH; osmolality 280–290 mOsm/L). After formation of a whole-cell configuration (–70 mV holding potential), current- or voltage-clamp protocols were applied. For the analysis of the firing pattern and current−voltage relationship, in current-clamp mode, a series of increasing currents (30-pA step, 500-ms duration with a 3-s interval) were injected. Evoked EPSCs were recorded from dentate gyrus granule cells (voltage-clamp conditions, –70 mV holding potential) by stimulation of glutamatergic afferents from perforant path using a bipolar theta capillary (2–5-μm tip) filled with ACSF and placed in the molecular layer of dentate gyrus. Stimuli were delivered at 0.33 Hz. Paired pulse ratio (PPR) was obtained (2nd EPSC/1st EPSC) by delivering paired pulses (20-, 50-, 75-, 100-, 150-, and 200-ms interstimulus intervals). AMPA/NMDA ratio was obtained (2nd EPSC at +40 mV/1st EPSC at –60 mV holding potential) by delivering paired pulses at 50-ms interstimulus intervals. Series and input resistances were monitored throughout the experiment using a –5 mV pulse. Recordings were considered stable when the series and input resistances, resting membrane potential, and stimulus artifact duration were not changed >20%. Cells that did not meet these criteria were discarded. The same procedure was conducted for electrophysiology recordings in 50-day-old AhR^f/f^ and AhR-icKO mice (4 weeks after 5 tamoxifen injections starting at 21 days old). Recordings were obtained by a PC-ONE amplifier, and signals were fed to a Pentium-based PC through a DigiData1322A interface board. pCLAMP 10.2 software was used for stimulus generation, data display, acquisition, storage, and analysis.

### Behavioral testing

#### Contextual fear conditioning

For mice, contextual fear conditioning occurred in test chambers (31 × 24 × 21 cm) with shock-grid floors. The front, top, and back of the chamber were clear acrylic, and the sides were modular aluminum. During training, AhR^–/–^ or AhR-icKO and their respective controls were placed in the chamber and, after 2 min of habituation, they received a mild or a weak conditioning protocol based on 3 foot shocks (0.6 mA, 2 s duration, 1 min apart) or 1 foot shock (0.48 mA, 2 s duration), respectively. After conditioning, mice were removed from the chamber 1 min after the last shock. Behavior was recorded by overhead cameras. Freezing (i.e., absence of movement except for breathing) was measured using automated scoring system for mice.

#### Barnes maze

A white circular platform (100-cm diameter, 70 cm above the floor) contained 20 holes equally spaced around its perimeter. Under one of the holes, there was an escape box (17 × 13 × 7 cm) filled with paper bedding. The location of this escape hole was always in the same place for all mice. For avoiding navigation based on olfactory or proximal cues within the maze, the platform was rotated before each trial, and the spatial location of the escape hole remained in a fixed location with respect to the distal room cues. During habituation, mice were allowed 5 min to freely explore the maze, with no escape box present. During training, mice were given 3 trials per day for 6 days. On each trial, the mouse was released in the center of the maze and allowed for 5 min to enter the escape box, where it remained for 30 s. If a mouse failed to find the escape box, it was guided by the experimenter. During probe test, the escape box was removed from the maze, and the mouse was allowed to search for 5 min. Time spent around each hole was recorded. Search paths were recorded by an overhead video camera and tracked using automated Ethowatcher software.

#### Novel object recognition (NOR) and novel object location (NOL)

Mice were placed in a rectangular arena (30 × 20 × 30 cm) with clear sidewalls containing two objects (A and B) for 8-min training session and returned to their home cages. The memory tests were performed as described (Nakashiba et al., 2008). Briefly, during the 8-min training phase of object recognition, two identical objects were placed in the arena. For the NOR test, the animal’s memory of one of the original objects was assessed by comparing the amount of time spent exploring a novel object compared with that spent exploring the familiar one during 8 min. For the NOL test, after the training period, the animal´s memory of one of the original objects was assessed by comparing the amount of time spent exploring the new located object with that spent exploring the original located one during 8 min. In both tests, the time spent exploring each object was expressed as a percentage of exploration time [% exploration time = (*t_novel_* – *t_familiar_*)/(*t_novel_* + *t_familiar_*)]. Behavior was recorded by overhead cameras, and videos analyses were performed by a blinded experimenter.

#### Y-maze

The Y-maze was made of three solid white arms of equal size (35 cm long and 5 cm wide with 10-cm-high walls) joined in a Y-configuration. The maze was cleaned with 70% ethanol between animals to eliminate traces of odor. For working memory, during habituation phase, mice were placed where the arms joined and allowed to freely explore the three arms for 6 min. Arm entry was defined as having forelimbs inside an arm. The number of entries was recorded to calculate the alternation percentage (defined as a set of consecutive arm entries), which was calculated by dividing the number of triads by the number of possible alternations multiplied by 100. For spatial memory, during training phase mice were allowed to explore two of the three arms for a total of 6 min while the third arm was blocked. Six hours later, mice were placed back in the maze for 6 min with all arms open, and the number of entries in each arm was recorded. Behavior was recorded by overhead cameras, and video analyses were performed by a blinded experimenter.

To avoid possible interference due to the manipulation, training, and testing on the histologic studies, we used different mouse cohorts for each set of experiments. Specifically, two separate mouse cohorts were used for both contextual fear conditioning and Barnes maze. For the rest of the behavioral testing, we used two different groups of mice. One group was first trained in NOR and 1 week later in NOL and, in the other one, mice were first tested on Y-maze and 1 week later in NOL.

### Statistical analysis

Results are expressed as mean ± SEM. Sample sizes for each experiment are indicated in the figure legends. In most experiments, sample size was estimated based on previous extensive experience with similar approaches. For specific experiments, we performed power analysis to adequate sample size with data from pilot studies (usually data from 3 animals/group) by calculating power analysis (http://www.biomath.info) with a significance level of 5% and ≥80% of power. Cohen’s *d* was used to calculate effect size, with observed strong size effects (*d* values) ≥ 0.8. Statistical significance was determined by use of a non-parametric, 2-tailed Mann–Whitney *t* test or a nonparametric, 2-way ANOVA followed by Bonferroni *post hoc* testing. Values of *p* < 0.05 were considered statistically significant. All statistical analyses were performed with Prism version 5.0 (GraphPad Software).

## Results

### Hippocampus-dependent memory is impaired in AhR^–/–^ mice

**Figure 1.**
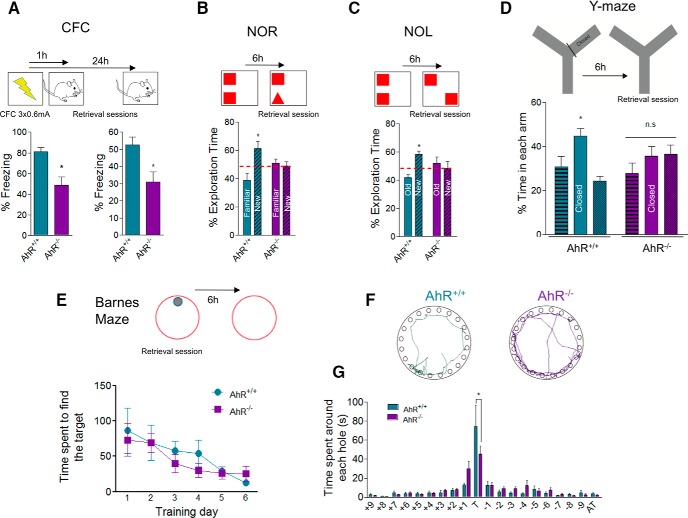
Ablation of AhR impairs hippocampus-dependent memory. ***A***, Percentage of freezing in the CFC task for AhR^+/+^ and AhR*^–/–^* mice 1 h (left) and 24 h (right) after the foot shocks (*, *p* < 0.05 versus AhR^+/+^; *n* = 8 AhR^+/+^ and 7 AhR*^–/–^* animals/group). ***B***, Percentage of exploration time between familiar and new object in the NOR test for AhR^+/+^ and AhR*^–/–^* mice 6 h after training. Two-way ANOVA demonstrated a significant interaction between the object and genotype [*F*_(1, 18)_ = 7,46; *p* < 0.05; *, *p* < 0.05 vs. AhR^+/+^; *n* = 6 AhR^+/+^ and 5 AhR*^–/–^* animals/group]. ***C***, Percentage of exploration time between old and new object location in the NOL test for AhR^+/+^ and AhR*^–/–^* mice 6 h after training. Two-way ANOVA demonstrated a significant interaction between the object and genotype [*F*_(1, 42)_ = 7.93; *p* < 0.05; *, *p* < 0.05 vs. AhR^+/+^; *n* = 12 AhR^+/+^ and 11 AhR*^–/–^* animals/group]. ***D***, Percentage of time spent in each arm in the Y-maze test for AhR^+/+^ and AhR*^–/–^* mice 6 h after training. Two-way ANOVA showed a significant interaction between the arm/genotype [*F*_(2, 27)_ = 3.84; *p* < 0.05; *n* = 6 animals/group]. ***E***, Time spent to find the escape box during the Barnes maze training sessions in both AhR^+/+^ and AhR^–/–^ mice. Two-way ANOVA demonstrated a significant effect during training sessions [*F*_(5, 65)_ = 5.04; *p* < 0.05; *n* = 7–8 animals/group]. ***F***, Density plots for grouped data showing where the AhR^+/+^ and AhR*^–/–^* mice concentrated their searches during retention test day. ***G***, Percentage of time (s) spent around each hole in the Barnes maze platform for AhR^+/+^ and AhR*^–/–^* mice during the retention test day. Two-way ANOVA demonstrated a significant interaction between the holes and genotype [*F*_(19, 260)_ = 1.83; *p* < 0.05; *, *p* < 0.05 vs. AhR^+/+^; *n* = 7–8 animals/group). Data are mean ± SEM. Data were compared by using nonparametric 2-tailed Mann–Whitney test (***A***), or a nonparametric 2-way ANOVA followed by Bonferroni *post hoc* testing (***B–G***).

To test whether AhR plays a role in hippocampus-dependent memory, 8-week-old AhR^–/–^ mice and their WT littermates were subjected to a battery of hippocampus-dependent tests. First, mice were trained in contextual fear conditioning (CFC), an associative learning which involves the hippocampus (Anagnostaras et al., 2001). No differences were found between WT and AhR^–/–^ mice in response to the foot shock (data not shown), indicating similar levels of nociception in both genotypes. During the retrieval, AhR^–/–^ mice displayed a reduced freezing response in both 1-h and 24-h tests (*p* < 0.05; Fig. 1*A*), and a decreased percentage of activity suppression (88.94 ± 3.52% vs. 61.50 ± 9.89% activity suppression in AhR^+/+^ vs. AhR^–/–^ mice, respectively, *p* < 0.05) compared with the control group, indicating a reduced fear memory in AhR^–/–^ mice, in agreement with previous evidence (Latchney et al., 2013). To confirm our results, we checked other types of hippocampus-dependent memory such as the novel object recognition (NOR) task that relies on mouse natural exploratory behavior (Ennaceur and Delacour, 1988). During the training session (two similar objects), no preference was detected for one object over the other in both genotypes (data not shown); however, during the test session, whereas WT mice presented a preferential exploration toward the new object, AhR^–/–^ mice exhibited impaired NOR performance with a lack of net preference for any of the objects (significant interaction between genotype and novel/old object recognition two-way ANOVA; *F*_(1,18)_ = 7.46; *p* = 0.0137; Fig. 1*B*). In addition, we checked whether spatial memory was also altered in AhR^–/–^ mice. First, mice were subjected to a novel object location task (NOL; Antunes and Biala, 2012), in which WT mice spent more time investigating the new location whereas, on the contrary, AhR^–/–^ mice explored similarly both locations (significant interaction between genotype and novel/familiar location exploration two-way ANOVA; *F*_(1,42)_ = 7.93; *p* = 0.0074; Fig. 1*C*). The impairment in spatial memory shown by AhR^–/–^ mice was corroborated by using the Y-maze and the Barnes maze. In the first one, no differences were observed in spatial working memory calculated either as percentage of spontaneous alternation (SAP) or as arm entries (% SAP: 63.14 ± 2.40 vs. 55.21 ± 2.81 in AhR^+/+^ vs. AhR^–/–^ mice, respectively, *p* > 0.05; arm entries: 26.43 ± 2.86 vs. 24.40 ± 5.39 in AhR^+/+^ vs. AhR^–/–^ mice, respectively *p* > 0.05; Fig. 1*D*) but, relative to WT, AhR^–/–^ mice did not show any preference toward the novel arm (two-way ANOVA; *F*_(2,27)_ = 3.84; *p* = 0.0342; Fig. 1*D*). In the Barnes maze (Fig. 1*E*), AhR KO mice and their WT littermates spent similar time to find the escape box during all training sessions. However, during the 24-h probe test, whereas WT animals spent most part of the time in the target hole, AhR^–/–^ ones did not (two-way ANOVA; *F*_(1.8,19)_ = 260; *p* = 0.0204; Fig. 1*G*). These results indicate impairment of the spatial memory and support that AhR is required for a proper function of hippocampus-dependent memory.

### Hippocampus-dependent memory deficits in AhR^–/–^ mice are independent of the levels of hippocampal neurogenesis

**Figure 2.**
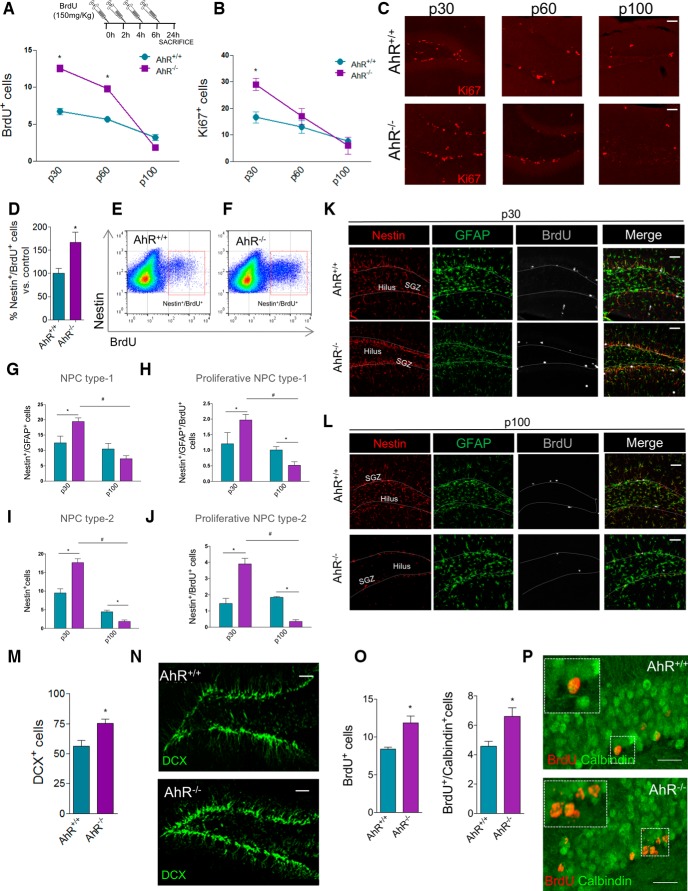
The absence of AhR exacerbates adult hippocampal neurogenesis. ***A***, BrdU^+^ cells in the DG of AhR^+/+^ and AhR^–/–^ mice measured at p30, p60, and p100 24 h after the last BrdU injection. Two-way ANOVA demonstrated a significant interaction between the age and genotype [*F*_(2, 24)_ = 9.51; *p* < 0.05; *, *p* < 0.05 vs. AhR^+/+^; *n* = 5 animals/group]. ***B***, ***C***, Ki67^+^ cells in the DG of AhR^+/+^ and AhR^–/–^ mice measured at p30, p60, and p100. Two-way ANOVA demonstrated a significant interaction between the age and genotype [*F*_(2, 28)_ = 8.56; *p* < 0.05; *, *p* < 0.05 vs. AhR^+/+^; *n* = 5–6 animals/group]. Representative images of Ki67^+^ cells in AhR^+/+^ and AhR^–/–^ mice at different time points are shown in ***C***. ***D–F***, Quantification of the number of nestin/BrdU^+^ cells by flow cytometry 24 h after BrdU administration. Data are expressed as the percentage of control group (*, *p* < 0.05 vs. AhR^+/+^; *n* = 6 animals/group; ***D***). Representative dot plots of double-stained cells for nestin and BrdU in the DG of AhR^+/+^ (***E***) and AhR^–/–^ (***F***) mice. ***G–L***, Characterization of type-1 (nestin^+^/GFAP^+^; ***G***) and type-2 progenitors (nestin^+^/GFAP^-^; ***I***) and their proliferative capacity (BrdU^+^; ***H*** and ***J***) in the DG of AhR^+/+^ and AhR^–/–^ mice determined at p30 and p100. Representative images of nestin (red), GFAP (green), BrdU (gray) of DG of AhR^+/+^ and AhR^–/–^ mice at p30 (***K***) and p100 (***L***; *, *p* < 0.05 vs. AhR*^+/+^*; #, *p* < 0.05 vs. AhR^–/–^; *n* = 4–6 animals/group). ***M***, ***N***, Quantification of the number of DCX^+^ cells in the DG of WT and AhR^–/–^ mice (*, *p* < 0.05 vs. AhR^+/+^; *n* = 7–8 animals/group). Representative images of DCX^+^ cells in AhR^+/+^ and AhR*^–/–^* mice are shown in ***N***. ***O***, ***P***, Quantification of the number of BrdU^+^ cells (left) and newborn integrated neurons (BrdU^+^/Calbindin^+^; right) determined 28 days after BrdU administration (*, *p* < 0.05 vs. AhR^+/+^; *n* = 7–9 animals/group). Representative colocalization images for BrdU and calbindin in AhR^+/+^and AhR^–/–^ mice are shown in ***P***. Insets display high-magnification images. Numbers of cells are expressed per 1000 μm^2^. Data are mean ± SEM. Scale bar is 50 μm in ***C***, ***J***, ***K***, and ***L***; 70 μm in ***N***; and 30 μm in ***P***. Data were compared by using nonparametric 2-tailed Mann–Whitney test in ***D***, ***M***, and ***O*** or nonparametric 2-way ANOVA followed by Bonferroni *post hoc* testing (***A***, ***B***, ***G–J***).

AhR-dependent impairment of hippocampal function in AhR^–/–^ mice could be due to a decrease in the levels of hippocampal neurogenesis, consistent with previous studies of AhR in this setting performed at 3 months (P90; Latchney et al., 2013). Interestingly, when animals were pulsed at different ages (p30, p60, and p100) with 4 consecutive BrdU injections (see Methods for details) at 2-h intervals and euthanized 24 h after the last injection, higher levels of proliferation at p30 and p60 but not at p100 were detected in AhR^–/–^ mice versus their WT littermates (Fig. 2*A*). The results at p30 and p100 were confirmed when we quantified the number of cells in cell cycle (using Ki67, marker of cells in active proliferation) in the SGZ of WT and AhR^–/–^ mice (Fig. 2*B*,*C*). Because memory studies had been performed at p60, deficits in proliferation are therefore unlikely to account for hippocampal deficits in AhR KO mice, though they may contribute to those reported previously at 3 months (P90; Latchney et al., 2013). Further confirming this finding, at p60, AhR^–/–^ mice displayed ∼60% increase in the number of proliferative nestin^+^ precursors (nestin^+^/BrdU^+^) in the DG compared with WT ones, determined by flow cytometry 24 h after BrdU administration (*p* < 0.05; Fig. 2*D–F*). Furthermore, these data were corroborated by analyzing both type-1 (nestin^+^/GFAP^+^) and type-2 (nestin^+^/GFAP^-^) neural progenitor cells, as well as their respective proliferation rates at p30 and p100 (Fig. 2*G–L*). At p30, AhR^–/–^ mice presented significantly higher levels of type-1 and -2 NPCs and its proliferative population than the control group but, although at p100 the population of type-1 cells was similar (*p* > 0.05; Fig. 2*G*), type-2 and both type-1 and type-2 proliferative AhR^–/–^ NPCs populations were dramatically decreased (*p* < 0.05, Fig. 2*G–J*) compared to WT populations.

To check whether the increase in SGZ proliferation induced by AhR absence was accompanied by a parallel increase in the number of adult newborn neurons, we first studied immunostaining of DCX, a marker of neuroblasts which is transiently expressed in new neurons. Supporting our previous data, p60 AhR^–/–^ mice displayed higher numbers of DCX^+^ cells, indicating immature neurons (*p* < 0.05, Fig. 2*M*). In addition, we administered BrdU during 5 days and, 28 days later, we analyzed the number of newborn mature neurons as BrdU^+^ cells expressing the mature neuronal marker calbindin. Quantifications demonstrated that AhR^–/–^ mice present a significant increase in the number of total BrdU^+^ cells and of double BrdU^+^/calbindin^+^ cells (*p* < 0.05; Fig. 2*O*), denoting that AhR absence leads to an increase in the number of newly generated neurons. Furthermore, a comparable percentage of BrdU^+^/calbindin^+^ cells was estimated for both groups (56% AhR^–/–^ vs. 55% WT), suggesting that AhR deletion does not modify differentiation rate.

Similarly, neurogenesis enhancement attributable to the absence of AhR, determined by quantification of BrdU^+^/nestin^+^ cells (4862 ± 561 vs. 8072 ± 851 cells in AhR^+/+^ vs. AhR^–/–^ mice, respectively, *p* < 0.05, *n* = 6), Ki67^+^ cells (276 ± 29 vs. 430 ± 23 cells in AhR^+/+^ vs. AhR^–/–^ mice, respectively, *p* < 0.05, *n* = 5) and DCX^+^ volume (3707 ± 364 vs. 6291 ± 545 μm^3^ in AhR^+/+^ vs. AhR^–/–^ p60 mice, respectively, *p* < 0.05, *n* = 5), was also observed in the other adult neurogenic niche, the subventricular zone (SVZ). Thus, a common shared mechanism underlying the actions of AhR in both neurogenic niches can be suggested.

Therefore, despite previously suggested role of AhR (Latchney et al., 2013), our data indicate that, at p30 and at p60, the absence of AhR enhances or does not affect hippocampal neurogenesis, thus discarding that hippocampus-dependent deficits observed in AhR^–/–^ mice at p60 are due to decreased levels of adult neurogenesis.

### Absence of AhR is associated with aberrant morphology of hippocampal granule cells

**Figure 3.**
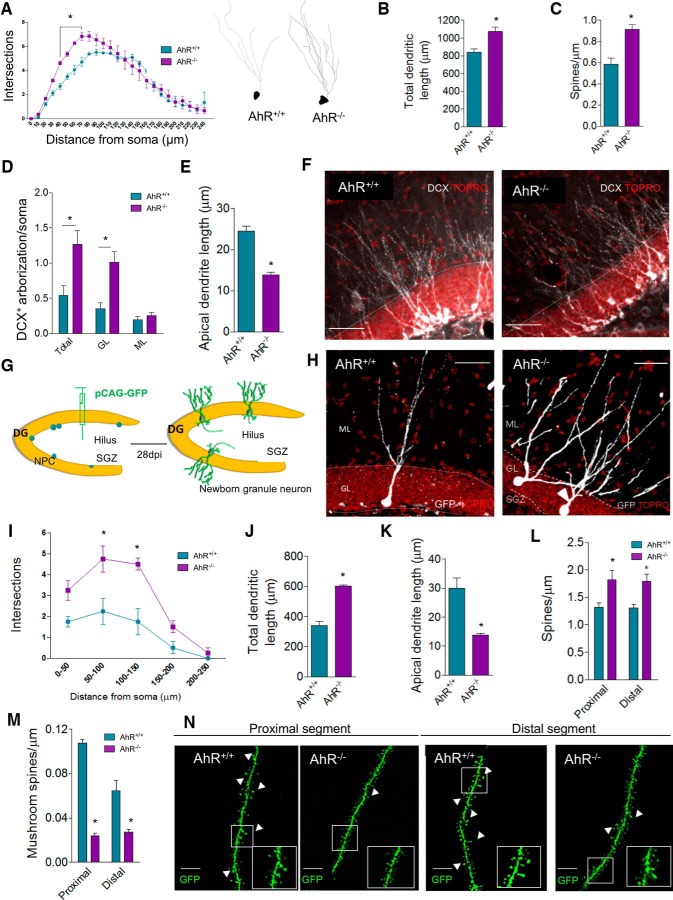
The absence of AhR alters granule cell morphology and dendritic spine density and maturation. ***A***, Sholl analysis of granule dendritic branching of Golgi-Cox stained DG from AhR^+/+^ and AhR^–/–^ p60 mice. Two-way ANOVA demonstrated a significant interaction between distance/genotype [*F*_(24,125)_ = 2.45; *p* < 0.05; *, *p* < 0.05 vs. AhR^+/+^; *n* = 23 WT and 37 AhR KO neurons from 3–4 animals/group]. Representative reconstructions of Golgi-Cox stained neurons are shown for AhR^+/+^ (left) and AhR^–/–^ mice (right). ***B***, Total dendritic length of Golgi-Cox-stained granular cells (*p < 0.05 versus AhR). ***C***, Quantification of spine density in Golgi–Cox stained neurons (*, *p* < 0.05 vs. AhR^+/+^; *n* = 20–30 dendrite segments from 3–4 animals/group). ***D***, Densitometric analysis of dendrite DCX^+^ labeling distribution in the DG of WT and AhR^–/–^ mice at p60. Data are displayed as the DCX^+^ integrated density found in total (GL+ML), GL, or ML normalized by values got from soma. ***E***, Quantification of apical neuroblast length. Representative images of DCX^+^ labeling distribution in AhR^+/+^ and AhR^–/–^ mice are shown in ***F*** (*, *p* < 0.05 vs. AhR^+/+^; *n* = 5–6 animals/group). ***G–N***, GFP-retroviral infection of newborn neurons. Schematic protocol followed for CAG-GFP retrovirus infusion in AhR^+/+^ and AhR^–/–^ p60 mice (***G***). Representative GFP newborn AhR^+/+^ (left) and AhR^–/–^ neurons (right) are shown in ***H***. Two-way ANOVA of Sholl analysis in GFP-labeled dentate granule cells demonstrates a significant interaction between the distance and genotype [*F*_(4,30)_ = 3.02; *p* < 0.05; *, *p* < 0.05 vs. AhR^+/+^; *n* = 19 WT and 28 AhR KO neurons from 4 animals/group; ***I***]. Quantification of total dendritic length (***J***) and apical dendritic length (***K***). Density of dendritic (***L***) and mushroom (***M***) spines in proximal and distal dendritic segments in the molecular layer of AhR^+/+^/GFP^+^ and AhR^–/–^/GFP^+^ granule cells 4 weeks postinfection (*, *p* < 0.05 vs. AhR^+/+^/GFP^+^; *n* = 26 and 22 segments from 4 animals/group). Representative images of GFP-labeled spines in both proximal and distal segments of AhR^+/+^/GFP^+^ and AhR^–/–^/GFP^+^ granule cells (N). Insets display high-magnification images. Data are mean ± SEM. Data were compared by using nonparametric Mann–Whitney tests in ***B–E*** and ***J–M*** or a nonparametric 2-way ANOVA followed by Bonferroni *post hoc* testing (***A*** and ***I***). Scale bar is 70 μm in ***F*** and ***H***, and 2 μm in ***N***.

A high rate of adult hippocampal neurogenesis has been previously demonstrated to correlate positively with increased learning and memory ability (Kempermann et al., 1997; van Praag et al., 2002). Given that AhR^–/–^ mice exhibit impaired memory when hippocampal neurogenesis is still enhanced and considering the high AhR mRNA expression in the DG granular layer (Kimura and Tohyama, 2017) and the reported role of AhR orthologues in the control of neuronal growth and dendritic arborization in invertebrates (Qin and Powell-Coffman, 2004; Crews and Brenman, 2006; Kim et al., 2006; Smith et al., 2013), we hypothesized that defects observed when AhR is deleted could be due to variations in the morphology of granule neurons and its subsequent functional activity. Therefore, we examined granule neuronal morphology in hippocampi of WT and AhR^–/–^ p60 mice. First, dendritic branching of DG granule neurons was quantified by Sholl analysis of Golgi-Cox stained sections (Fig. 3*A–C*). Relative to WT, AhR^–/–^ mice displayed a higher number of dendritic branches close to the soma (40–70 µm from soma; *p* < 0.05; Fig. 3*A*), increased total dendritic length (*p* < 0.05; Fig. 3*B*), and a larger number of dendritic spines (*p* < 0.05; Fig. 3*C*), supporting our hypothesis that the absence of AhR is associated with an altered dendritic structure of granule neurons.

Interestingly, similar changes were found in immature neurons (DCX^+^ neuroblasts) in WT and AhR^–/–^ p60 mice, indicating that AhR effects on granule neuronal morphology seem to be also apparent in adult-born granule neurons. Interestingly, quantification of DCX^+^ labeling distribution between total (GL+ML), granular (GL) and molecular layers (ML) revealed significant differences in the arborization pattern of both genotypes: compared with WT subjects, AhR^–/–^ mice showed increased DCX^+^ staining in GL while no differences were found in the ML (*p* < 0.05; Fig. 3*D*). In addition, immature AhR KO cells also presented a significant reduction in the apical dendrite length (*p* < 0.05; Fig. 3*E*). To confirm that the absence of AhR affects the morphology of adult newborn neurons, high titers of CAG-GFP retrovirus were delivered into the hilar region of WT and AhR^–/–^ KO p60 mice to selectively target proliferating neuronal progenitors *in vivo* (Zhao et al., 2006). 28 days postinfection, Sholl analysis of GFP newborn neurons in the DG showed an increased dendritic branching in the proximal segment close to the soma of AhR^–/–^ neurons compared to those of WT (50–150 µm from the soma; *p* < 0.05; Fig. 3*I*). While total dendritic length was significantly greater in AhR^–/–^/GFP^+^ neurons (*p* < 0.05; Fig. 3*J*), indicating a higher pattern of branching, the apical dendrite length was shorter than in control/GFP^+^ cells (*p* < 0.05; Fig. 3*K*). Taking all these results together, it can be concluded that the absence of AhR promotes retraction of the apical dendrite and altered branching of the dendritic tree. The aberrant morphology of granular neurons was additionally supported by the finding of a higher dendritic spine density in AhR^–/–^/GFP^+^ neurons compared to those of WT mice in both proximal and distal segments of dendritic branches in the molecular layer (*p* < 0.05; Fig. 3*L*). Despite this, the number of mushroom spines in both segments, typically more abundant in mature neurons (Zhao et al., 2006), was significantly lower in AhR^–/–^/GFP^+^ cells than in AhR^+/+^/GFP^+^ neurons along the dendrites in the molecular layer (*p* < 0.05; Fig. 3*M*), thus suggesting a more immature phenotype in the absence of AhR.

### The lack of AhR alters granule cell intrinsic excitability, synapse maturation, and the correct function of the hippocampus

**Figure 4.**
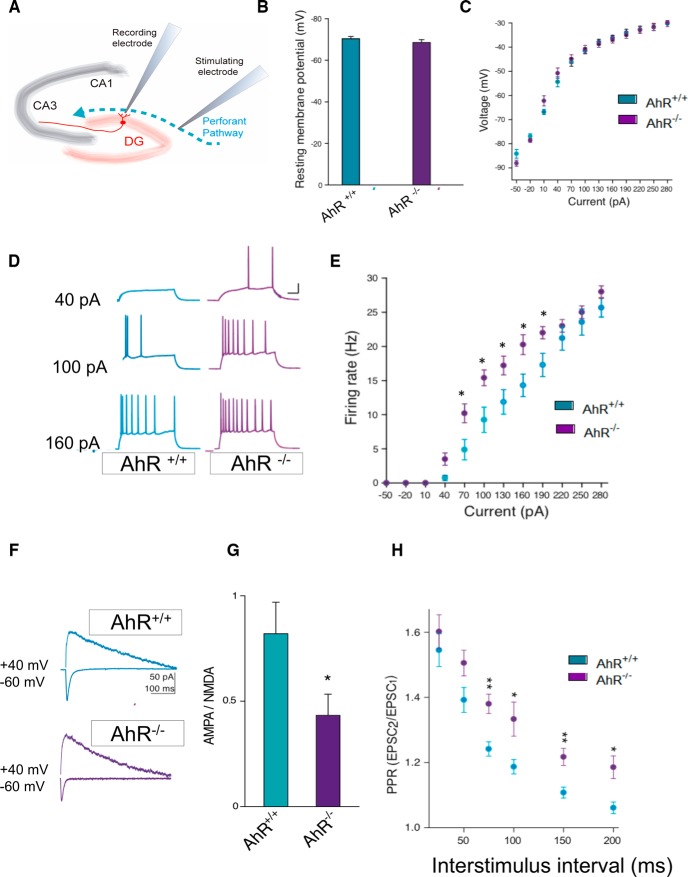
AhR deletion alters the synaptic properties of dentate gyrus granule cells. ***A***, Schematic representation of a hippocampal slice showing stimulating and recording electrode sites. ***B***, ***C***, The resting membrane potential (***B***) and the current−voltage relationship (***C***) were not significantly different between DG granule cells from AhR^+/+^ and AhR^–/–^ mice (*n* = 4 animals/group). ***D***, ***E***, DG granule cell firing rate is significantly increased by AhR deletion (*, *p* < 0.05 vs. AhR^+/+^). Representative sample traces (***D***) and averaged values (***E***) in response to increasing depolarizing currents. ***F***, ***G***, AMPA/NMDA ratio is decreased by AhR deletion (*, *p* < 0.05 vs. AhR^+/+^). Representative traces (***F***) and averaged values (***G***) of NMDA- and AMPA-mediated EPSCs recorded at +40 and –60 mV, respectively. ***H***, Averaged values showing a significant increase in the PPR at interstimulus intervals of 75, 100, 150, and 200 ms in cells lacking AhR (*, *p* < 0.05, and **, *p* < 0.01 vs. AhR^+/+^). EPSC sample traces represent the mean of 20 consecutive EPSCs at 0.33 Hz. Data are mean ± SEM (14 neurons from 8 slices from *n* = 4 AhR^+/+^ mice and 12 neurons from 8 slices from *n* = 4 AhR^–/–^ mice). Data were compared by using nonparametric 2-tailed Mann–Whitney tests.

Morphologic changes resulting from AhR ablation can lead to alterations of the hippocampal physiologic properties. This possibility was investigated by performing electrophysiological analysis of granule cells in acute slices from AhR^–/–^ and control mice (Fig. 4*A*). Previously, the absence of significant differences between groups in resting membrane potential and membrane resistance was verified (Fig. 4*B*,*C*). Then, the ability of AhR^–/–^ granule cells to fire repetitive action potentials, a hallmark of neuronal maturation (Deng et al., 2010), was assessed. Under the whole-cell current-clamp, AhR^–/–^ granule cells showed an increased firing rate in response to a depolarizing current injection (40–190 pA; *p* < 0.05; Fig. 4*D*,*E*), consistent with immature neuronal excitability (Dieni et al., 2016). It is known that the ratio of active/silent synapses changes over neuron maturation as a result of changes in the content of glutamate AMPA receptors (AMPARs; Carlisle and Kennedy, 2005; Tada and Sheng, 2006; Paoletti et al., 2013; Chater and Goda, 2014). To assess functional AMPAR content, we quantified the AMPAR/NMDAR ratio by comparing AMPA excitatory postsynaptic currents (EPSCs) at –70 mV and glutamate NMDA receptor (NMDAR) EPSCs at +40 mV in WT and AhR^–/–^ granule cells. We found a significantly lower AMPAR/NMDAR ratio in AhR^–/–^ granule cells, indicating a lower proportion of AMPAR to NMDAR on their granule cell dendrites (*p* < 0.05; Fig. 4*F*,*G*), thus suggesting that AhR^–/–^ granule cells have a more immature phenotype than those from WT. Finally, we studied the pair-pulse ratio (PPR) as a hippocampus-dependent function measurement for integration of inputs coming from the entorhinal cortex to the hippocampal granule cells. The PPR (EPSC2/EPSC1) was significantly higher in AhR^–/–^ KO compared with control mice (*p* < 0.05; Fig. 4*H*), indicating that the initial probability of release is lower in AhR^–/–^ KO mice.

### Absence of AhR in neural progenitor cells is enough to promote hippocampal deficits

**Figure 5.**
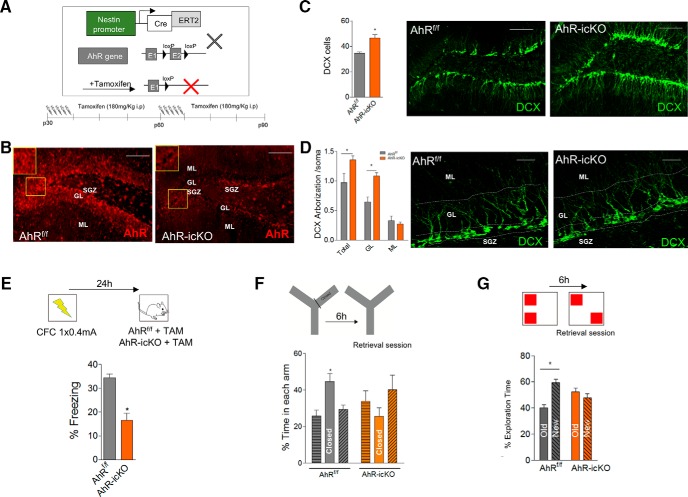
Acute ablation of AhR in adult neural precursors impairs hippocampus-dependent memory by promoting aberrant immature neurons. ***A***, Schematic diagram of the strategy for conditional deletion of AhR in NPCs in nestin-Cre^ERT2+/^AhR^f/f^ mice (AhR-icKO). AhR^f/f^ and nestin-Cre^ERT2+/^AhR^f/f^ mice were administered two rounds of tamoxifen (TAM; at p30 and p60) at a dose of 180 mg/kg. ***B***, AhR immunostaining in AhR^f/f^ (left) and AhR-icKO (right) 3 weeks after the second round of TAM injection. ***C***, Quantification of the number of DCX^+^ cells per 1000 μm^2^ in the DG of AhR^f/f^ and AhR-icKO mice 3 weeks after the last TAM injection (*, *p* < 0.05 versus AhR^f/f^; *n* = 8 animals/group; left). Representative images of DCX^+^ staining in AhR^f/f^ and AhR-icKO mice (right). ***D***, Densitometric analysis of dendrite DCX^+^ labeling distribution in the DG of AhR^f/f^ and AhR-icKO mice 3 weeks after the last TAM injection. Data are displayed as the DCX^+^ integrated density found in total (GL+ML), GL, or ML normalized by values got from soma (*, *p* < 0.05 vs. AhR^f/f^; *n* = 6 AhR^f/f^ and 5 AhR-icKO animals/group; left). Representative images of the arborization of immature newborn cells in AhR^f/f^ and AhR-icKO mice (right). ***E***, Protocol followed for a weak contextual fear conditioning paradigm (0.4 mA ×1) in AhR^f/f^ and AhR-icKO treated with tamoxifen. Retrieval was performed 24 h after training. (*, *p* < 0.05 vs. AhR^f/f^; *n* = 10–11 animals/group). ***F***, Percentage of time spent in each arm in the Y-maze test for AhR^f/f^ and AhR-icKO mice 3 weeks after the last TAM injection 6 h after training. Two-way ANOVA demonstrated a significant interaction between the arm and genotype [*F*_(2, 57)_ = 5.91; *p* < 0.05; *n* = 10–11 animals/group). ***G***, Percentage of exploration time between old and new object location in the NOL test for AhR^f/f^ and AhR-icKO mice 3 weeks after the last TAM injection 6 h after training (*n* = 10–11 animals/group). Data are mean ± SEM. Scale bar is 50 μm in ***B–D***. Data were compared by using nonparametric 2-tailed Mann–Whitney test (***C–E***), or nonparametric 2-way ANOVA followed by Bonferroni *post hoc* testing (***F***, ***G***).

To check the specific role of AhR in adult-born granule neurons, we used a transgenic strategy to conditionally delete AhR from neural progenitor cells in adult mice (AhR-icKO mice; Fig. 5*A*). For such purpose, we crossed mice expressing tamoxifen (TAM)-inducible Cre-recombinase driven by a progenitor specific promoter (nestin-Cre^ERT2^ mice; Imayoshi et al., 2008) with mice in which AhR is floxed (AhR^f/f^; Walisser et al., 2005). In double mutant offspring from this cross (AhR-icKO), TAM treatment (Fig. 5*A*, bottom) induced the excision of the exon 2 flanked sequences, and the deletion of AhR in neural progenitor cells and their progeny resulted in a marked decrease of AhR expression in the DG determined by immunofluorescence (Fig. 5*B*). Examination of 3-month-old animals after tamoxifen administration revealed a higher number of DCX^+^ cells in the SGZ of the AhR*-*icKO mice than in that of control mice (*p* < 0.05; Fig. 5*C*), although no significant differences were found for Ki67^+^ cells (29.28 ± 2.93 vs. 33.47 ± 1.86 in AhR^+/+^ vs. AhR^–/–^ mice, respectively; *p* > 0.05). Consistent with these data, AhR*-*icKO mice treated with TAM presented an increased DCX staining in total (GL+ML) and GL but not in the ML (*p* < 0.05; Fig. 5*D*), similar to AhR^–/–^ mice. To study whether morphologic alterations present in newborn neurons were affecting cognitive ability, memory tests were conducted. First, we analyzed episodic memory in CFC test using a weak fear conditioning protocol based on one-foot shock stimuli to detect subtle differences that are not masked by generalization. Remarkably, in agreement with our results in AhR^–/–^ mice, AhR*-*icKO mice displayed comparable cognitive hippocampal deficits in CFC, showing a reduced freezing response compared to AhR^f/f^ (*p* < 0.05; Fig. 5*E*). Similarly, in the Y-maze test, only AhR^f/f^ mice showed a preference toward the closed arm (significant interaction between genotype and closed arm two-way ANOVA; *F*_(2, 57)_ = 5,91; *p* = 0.0047; Fig. 5*F*). Finally, NOL test revealed that the specific ablation of AhR in adult neuroprogenitor cells in AhR-icKO mice pattern separation skills is altered, showing a worst discrimination efficiency than AhR^f/f^ mice, not demonstrating any kind of preference for the new location (Fig. 5*G*).

### Acute conditional deletion of AhR in neuroprogenitor cells is enough to alter granule cell intrinsic excitability, synapse maturation, and the correct function of the hippocampus

**Figure 6.**
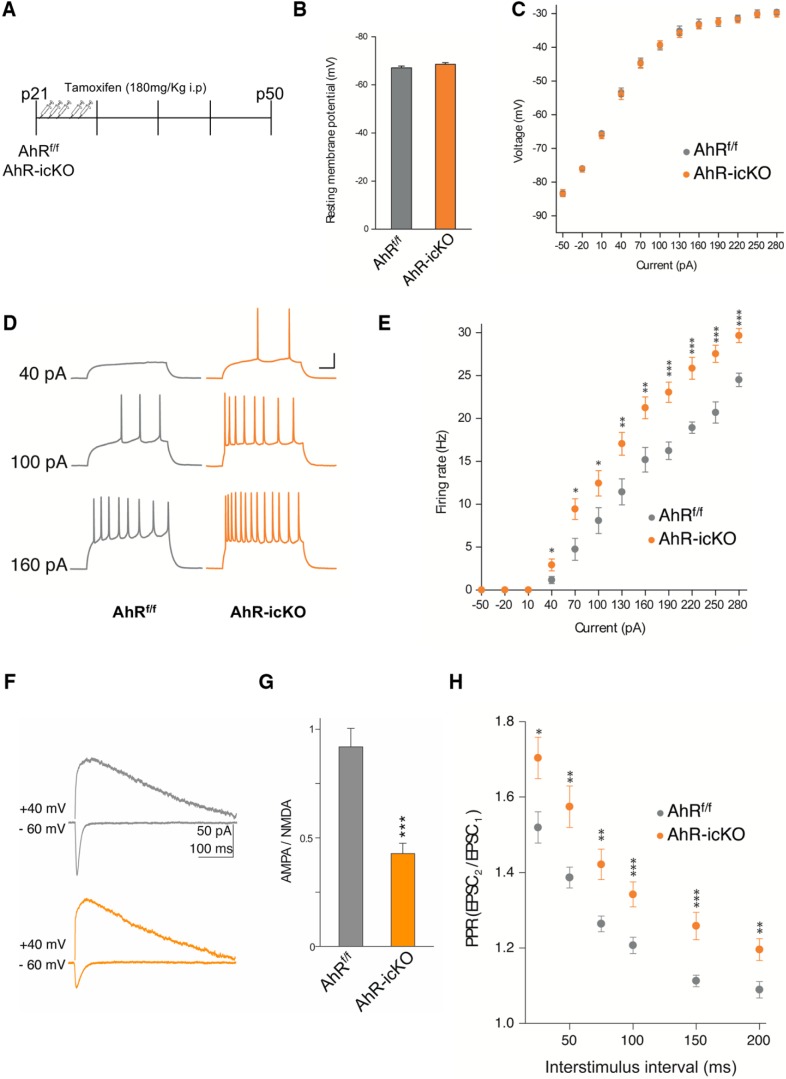
Acute ablation of AhR in adult neural precursors alters the synaptic properties of dentate gyrus granule cells. ***A***, Experimental protocol for tamoxifen administration and electrophysiological recordings. ***B***, ***C***, The resting membrane potential (***B***) and the current−voltage relationship (***C***) did not show significant differences between DG granule cells from AhR^f/f^ and AhR-icKO mice (*n* = 4–6 animals/group). ***D***, ***E***, DG granule cell firing rate is significantly increased by specific AhR ablation (*, *p* < 0.05 vs. AhR^f/f^). Representative sample traces (***D***) and averaged values (***E***) in response to increasing depolarizing currents. ***F***, ***G***, AMPA/NMDA ratio is decreased by AhR deletion. Representative traces (***F***) and averaged values (***G***) of NMDA- and AMPA-mediated EPSCs recorded at +40 and –60 mV, respectively (*, *p* < 0.05, **, *p* < 0.01, and ***, *p* < 0.001 vs. AhR^f/f^). ***H***, Averaged values showing a significant increase in the PPR at interstimulus 75, 100, 150, and 200-ms intervals in cells lacking AhR (*, *p* < 0.05, **, *p* < 0.01, and ***, *p* < 0.001 vs. AhR^f/f^). Data are mean ± SEM (25 neurons from 8 slices from *n* = 6 AhR^f/f^ mice and 24 neurons from 8 slices from *n* = 4 AhR-icKO mice). Data were compared by using nonparametric 2-tailed Mann–Whitney tests.

To examine whether the functional properties of the aberrant newborn neurons might underlie hippocampus-dependent memory impairments observed in our AhR-icKO, we acutely ablated AhR in adult neuroprogenitor cells by treating p21 AhR^f/f^ and AhR-icKO mice with tamoxifen, and electrophysiological studies were performed 4 weeks after the last tamoxifen administration (Fig. 6*A*). We did not see any differences between groups in resting potential and membrane resistance (Fig. 6*B*,*C*). When we analyzed the intrinsic excitability of AhR^f/f^ and AhR-icKO granule neurons, measured as the ability to spike action potentials by stimulating them with increased electrical depolarization currents, we found that acute ablation of AhR in adult neuroprogenitor cells led to a significant increase in the intrinsic excitability of AhR-icKO granule neurons (*p* < 0.05; Fig. 6*D*,*E*), consistent with our previous results in constitutive AhR KO mice. Given the importance of AMPA receptors in maturation and functionality of the synapses, AMPAR- and NMDAR-mediated currents were measured to explore the ability to integrate and respond to the inputs coming from the entorhinal cortex into the hippocampus by the granule neurons. Confirming our results in AhR^–/–^, AhR-icKO granule neurons displayed a reduction in AMPA/NMDA ratio, indicating that the specific absence of AhR in newborn granule neurons impairs a correct dendritic spine maturation (*p* < 0.05; Fig. 6*F*,*G*). Finally, we studied the PPR in these mice to explore the connectivity between EC and DG. In accordance with our previous results in AhR KO mice, AhR-icKO granule neurons showed significant higher PPR (EPSC2/EPSC1) compared with AhR^f/f^ cells (Fig. 6*H*), meaning an initial lower neurotransmitter probability of release when AhR is acutely ablate.

## Discussion

We have investigated the role of the transcription factor AhR in hippocampal function and granule neuronal morphology in the adult mouse brain. Our data demonstrate that AhR absence is associated with a severe impairment of hippocampal-dependent memory, concomitantly with an increased dendrite arborization pattern and a decreased dendritic spine maturity of hippocampal granule neurons, as well as aberrant electrophysiological properties and functions of these cells.

AhR (aryl hydrocarbon receptor) is a basic helix-loop-helix-Per-ARNT-Sim (bHLH-PAS) transcription factor that mediates the toxic and carcinogenic effects of xenobiotics. Interestingly, AhR is widely expressed in the CNS and, in this context, its physiologic and pathologic roles are just beginning to be unraveled. Specifically, AhR mRNA is enriched in the dentate gyrus granule cells of the adult hippocampus (Kimura and Tohyama, 2017), a crucial structure for high-cognition tasks, such as episodic learning, spatial learning, and memory, which are often disrupted in neurologic disorders. AhR is the only PAS member known to be activated by endogenous or exogenous ligands (Mimura et al., 1997; Mimura and Fujii-Kuriyama, 2003; Mandal, 2005; Nguyen and Bradfield, 2008; Boitano et al., 2010; Cuartero et al., 2014). The potential therapeutic implications derived from this fact prompted us to study its role in hippocampal function. Here, we demonstrate that the absence of AhR affects different types of hippocampal-dependent tasks such as episodic (NOR and CFC) and spatial memories (Y-maze, NOL, and Barnes maze).

Hippocampal newborn neurons have been implicated in the acquisition and recall of hippocampus-dependent memories (Aimone et al., 2014; Anacker and Hen, 2017) and, therefore, adult hippocampal neurogenesis is an essential process for cognitive function (Kempermann et al., 1997; van Praag et al., 1999, 2002; Zhao et al., 2006). The reduced NPC proliferation in the SGZ in parallel to deficits in fear conditioning memory tests that has been described in 12-week-old AhR-deficient mice (Latchney et al., 2013) could therefore explain the AhR-dependent impairment of episodic and spatial hippocampus-dependent memory mice that we observe in 2-month-old (p60) AhR^–/–^. However, on the contrary, we have found that 2-month-old (p60) AhR^–/–^ mice display enhanced SGZ neurogenesis, as shown by a higher number of newborn NPCs, neuroblasts, and fully integrated mature neurons, thus discarding that hippocampus-dependent memory deficits are due to decreased adult neurogenesis. Indeed, we have found that at both p30 and p60, the absence of AhR increases SGZ NPC proliferation. However, at p100, the process is reversed, with a decrease in NPC proliferation, similarly to the reported results at 12 weeks old (Latchney et al., 2013). It is plausible that niche depletion induced by the enhanced NPC proliferation at earlier times accounts for the decreased proliferation at later times in AhR^–/–^ mice. Several studies have reported an increased proliferative response, so-called compensatory proliferation, that takes place to counteract an excess of newborn cell death (Yamaguchi and Miura, 2015). Although the parallel increase in the number of BrdU^+^/calbindin^+^ cells suggests that this mechanism is not taking place in AhR^–/–^ mice, additional studies assessing precursor apoptosis and survival would be required to define the outcomes of excess proliferation in the absence of AhR.

In any case, as discussed above, decreased proliferation is not responsible for hippocampal memory deficits in 2-month-old mice, supporting that other mechanisms are involved.

AhR is a highly conserved protein from invertebrates to mammals. Although in mammals AhR has been traditionally known to participate in the xenobiotic metabolism of toxic compounds like dioxins (Fernandez-Salguero et al., 1996, 1997; Fujii-Kuriyama and Mimura, 2005; Barouki et al., 2007; Fujii-Kuriyama and Kawajiri, 2010), invertebrate AhR orthologues do not have a toxic response to dioxin, and neither do they have dioxin-binding capacity, what suggests another ancestral role for this receptor that could have remained throughout evolution. In fact, previous works in *Caenorhabditis elegans* (Huang et al., 2004; Qin and Powell-Coffman, 2004; Smith et al., 2013) and *Drosophila* (Crews and Brenman, 2006; Kim et al., 2006) identified AhR orthologues as regulators of dendrite branching in different types of neurons. More recently, in mammals, AhR activation has been reported to disrupt migration and dendritic growth of olfactory interneurons and hippocampal CA1 neurons, respectively, in the mouse brain (Kimura et al., 2016, 2017). However, the specific role of AhR in dendrite morphology and functional activity of DG granule neurons in the adult murine brain is not known. Our studies using Golgi–Cox staining or doublecortin immunofluorescence show altered dendritic structure of granule neurons in the absence of AhR. GFP labeling confirmed that AhR absence affects the morphology of adult newborn neurons, an effect that likely contributes to the memory deficits exhibited by these mice. AhR^–/–^ newborn neurons show an altered morphology characterized by a shorter apical dendrite and a profuse dendritic branching close to the soma. Because ectopic migration was not observed, the shortening of the apical dendrite in AhR^–/–^ cannot be ascribed to an ectopic pattern of migration in the GL. All these changes could affect the synaptic partners of these neurons, with detrimental consequences for hippocampal-dependent behavior, and suggest that modifications of AhR function might underlie some pathologic situations inducing aberrant granule neuronal morphology.

Regarding the morphology of dendritic spines, whereas filopodia and stubby spines are often associated with immature neurons, thin and mushroom spines are more abundant in mature neurons (Carlisle and Kennedy, 2005; Zhao et al., 2006; Spruston and Johnston, 2008). Of note, GFP labeling revealed that AhR^–/–^ granule neurons displayed a higher spine density but a much lower abundance of mature mushroom spines. Thus, reduced dendritic mushroom spine density would further alter excitatory inputs and the number of synaptic inputs and integration (Spruston and Johnston, 2008).

Consistently, AhR loss of function was associated with an increased intrinsic excitability which could suggest a more immature phenotype (Dieni et al., 2016; Lopez-Rojas and Kreutz, 2016). Previous studies have reported that neuronal membrane resistance as well action potential firing rate decrease along neuronal maturation (Mongiat et al., 2009; Dieni et al., 2016), resulting in an adequate integration of the inputs coming from the entorhinal cortex. These changes are critical factors that contribute to learning and memory in the hippocampus. Our results suggest that AhR could be necessary for this electrophysiological change. We also found a lower AMPAR/NMDAR ratio in granule neurons from AhR^–/–^ mice, probably indicating a selective depression in AMPAR synaptic responses. During physiologic granule cell maturation, there is an increase in the number of mature dendritic spines due to a progressive incorporation of AMPARs into the synaptic sites (Bassani et al., 2013). As commented above, AhR deficiency was linked to a higher proportion of immature spines, albeit an increased spine density in mature granule cells. Our results support the idea that AhR is necessary for this maturation, very likely by modulating the levels of AMPAR and NMDAR in the dendritic spines. In addition, the PPR (EPSC2/EPSC1) was significantly higher in AhR^–/–^ hippocampal slices compared with those from wild-type mice, strongly suggesting that the initial probability of neurotransmitter release in the terminals coming from the entorhinal cortex to the dentate gyrus is reduced in AhR^–/–^ mice (Fioravante and Regehr, 2011). This could suggest an immature phenotype of granule cell dendritic spines that affects synapses in a retrograde fashion, by altering neurotransmitter release from perforant pathway terminals.

In contrast with our results, increased rates of neurogenesis and/or high excitability on immature newborn granule cells are two features that have been associated with the role of these cells in learning and memory (Lopez-Rojas and Kreutz, 2016). However, several pieces of evidence in the literature also support that increased neurogenesis may not always result in improved function. For instance, manipulations that increase neurogenesis may have positive effects on anterograde memories but not in retrograde memories (Frankland et al., 2013; Akers et al., 2014). In agreement with our results, pathologic situations that impair hippocampal function such as epilepsy (Zhao et al., 2008; Cho et al., 2015) or stroke (Niv et al., 2012; Woitke et al., 2017) trigger an augmented hippocampal neurogenic response which is accompanied by aberrant features of newborn neurons. Of note, these aberrant neurons show a retraction in apical dendrite and an increased aberrant dendrite branching, a phenotype which is considered an immature feature (Woitke et al., 2017) and clearly resembles the one that we have observed in AhR^–/–^ mice. This altered dendritic morphology may, in its turn, underlie the “immature” electrophysiological features described. As commented above, an interesting possibility suggested by our study is that AhR controls dendritic spine formation and maturation, very likely by regulation of the expression and/or posttranslational trafficking of the AMPA receptor, a hypothesis that remains to be studied. A sustained immature status due to the lack of proper dendritic maturation could trigger, as compensatory mechanisms, an intrinsic hyperexcitability and a subsequent lower initial probability of release, that will translate into an increased PPR. Although the underlying mechanisms are likely to differ depending on each pathophysiological circumstance, it has been described that animal paradigms of Alzheimer’s disease (AD) show aberrant increases in network excitability in the dentate gyrus hippocampus that may contribute to the neurologic deficits shown by these models (Palop et al., 2007; Hazra et al., 2013; Frazzini et al., 2016).

To further confirm the role of AhR in hippocampus-dependent memory through the modulation of neurogenesis and granule neurons maturation and activity, we analyzed those mechanisms in our specific neuroprogenitors AhR-ablated mice. Of note, we evaluated SGZ NPC proliferation using animals with specific AhR deletion in nestin-expressing cells. Our data showing higher numbers of immature neurons in the SGZ of AhR icKO mice, and altered dendritic arborization and electrophysiological properties similar to those of AhR^–/–^ mice, confirm our previous data and allow us to discard indirect effects arising from the total lack of AhR in AhR^–/–^ mice. Besides, similar cognitive impairment was observed after the specific deletion of AhR in neuroprogenitor cells. First, by using a weak protocol in the CFC test, our data showed that the absence of AhR in newborn neurons is enough to disturb episodic memory. Furthermore, similar memory impairment was observed in AhR-icKO mice when they were tested on the Y-maze, NOR and NOL tests, supporting that alteration of the AhR pathway in newborn granule cells, or even changes in the availability of endogenous or exogenous AhR activators, not only may affect their morphologic properties, but also could have detrimental repercussions in the function of the hippocampus.

Like several other members of the bHLH superfamily and consistent with its dynamic pattern expression in the embryonic and postnatal mouse brain at different time points (Kimura and Tohyama, 2017), brain AhR is likely to play both time- and region-specific functions. In agreement with this, it has been shown that AhR activity regulates cerebellar granule neuron number and differentiation, possibly by coordinating granule neuron precursor developmental transition (Dever et al., 2016). Although with different features, our longitudinal study illustrates how hippocampal neurogenesis varies along time from p30 to p100, strongly supporting a role of AhR in the control of proliferation and pool maintenance of adult-hippocampal neural stem cells in an age-dependent way.

In addition, several studies report the involvement of AhR in the migration and dendritogenesis of olfactory interneurons and cortical pyramidal neurons during development (Kimura et al., 2016, 2017), as well as in neuronal differentiation in adult mice (Dever et al., 2016). In line with these results, in the present study our data demonstrate the role of AhR as a key modulator of newborn hippocampal granule neuron morphology, including dendritic spine growth and maturation, which are essential for hippocampal function, thus supporting AhR as a main player in neuronal maturation/differentiation. Hence, our study expands the biological implications of AhR receptor in such a relevant function showing that AhR plays time-specific functions in the regulation of neurogenesis and granule neuron maturation in hippocampus. Interestingly, several studies have demonstrated that dioxin exposure interferes with developmental neurogenesis (Williamson et al., 2005; Latchney et al., 2013), so abnormal modulation of AhR by ligands such as environmental pollutants throughout life might lead to hippocampus-related dysfunctions.

Summing up, deletion of AhR induces a severe impairment of hippocampal-dependent memory, concomitantly with a high dendrite arborization pattern in newborn hippocampal neurons, increased density of dendritic spines, but a reduction in mature mushroom spines, which drive a phenotype showing aberrant electrophysiological properties and function of these cells. Our study provides evidence indicating that AhR is a regulator of dendrite arborization and functional activity of adult hippocampal newborn neurons and showing its critical role for learning and memory function. An additional novel line of research opened by our study is the possible implication of AhR, as a new potent druggable target, in disorders in which cognitive deficits are accompanied by hippocampal morphologic alterations, such as epilepsy, schizophrenia, or stroke.

## Synthesis

Reviewing Editor: Mike Fainzilber, Weizmann Institute of Science Decisions are customarily a result of the Reviewing Editor and the peer reviewers coming together and discussing their recommendations until a consensus is reached. When revisions are invited, a fact-based synthesis statement explaining their decision and outlining what is needed to prepare a revision will be listed below. The following reviewer(s) agreed to reveal their identity: Michael Kreutz, Emanuel DiCicco-Bloom.

The authors have satisfactorily addressed the majority of concerns, improving the clarity of the Methods and the overall MS. A few issues remain as below.

The authors might consider revising/replacing the term “integration,” as used in the Title, Abstract, Significance, and Introduction. To this reviewer the word connotes the insertion of newborn neurons into the DG Granule Cell Layer (GCL), and their establishment of neural connections. In fact, the authors use as an example of failed neuron integration, epilepsy, where the excessive neurogenesis leads to more newborn cells, as well as misplaced/ectopic neurons that do not properly integrate into GCL, but localize in the wrong places. The authors identify in the AhR-/- mice excess proliferation, proper neuron localization but abnormal dendrite formation, excess spine density but fewer mushroom spines, and associated abnormal neurophysiological properties. Thus neuron morphogenesis/cytoarchitecture and synaptic activity are abnormal in AhR-/- mice, which impacts neurons and circuits/connectivity, which changes do not seem to be captured by the term integration. Use of the term may appear misleading or non-specific; it is not an additional abnormal property, but an imprecise descriptor, from this point of view.

The authors have clarified their methods and improved rigor of data quantification, that shows increased neural precursor proliferation that occurs early in life (P30, P60) but ceases at later ages (P100). It is perhaps surprising that they do not quantify actual neurogenesis, which is the product of both proliferation as well as cell survival/death. How many GCL neurons are there at the various ages? It may well be the case that there are increases in absolute numbers at early ages, or alternatively, deficiencies at all ages, especially if excess proliferation of early precursors that express nestin, is a compensation in response to excess death of immature neurons that express DCX, or postmitotic neurons. This pattern of excess precursor proliferation as a compensation of DCX+ cell death has been reported in multiple genetic models of neurodevelopmental disorders. While the authors do not need to actually perform stereological assessment of GCL neuron numbers, they should discuss that the outcomes of excess proliferation remain to be defined in this model, and that precursor apoptosis, survival, and neuron cell counts would address this issue.

Methods: Page 7, line 132: Surprisingly, they state that “Animals of both sexes were used ...” Please address. This now contradicts what is said in Point #7 in the Rebuttal!

Page 8, line 164: Please drop the word “mouse” from this phrase: “rabbit anti-mouse Ki67 (nuclear protein specifically expressed in cells undergoing active proliferation; 1:500, Abcam).”

Page 9, lines 186-191: The authors have provided correction of the 1:5 series (not 1:10), and now express cell number per 1000µm2. And they indicate that the fixed use of 5 sections “almost cover the dorsal hippocampus” (Rebuttal). The authors state: “We believe that, in this way, we are correcting a possible bias caused by the estimation of cell numbers in different DG areas.” This is not correct, however. Expressing cell number by cell region area is certainly better than simply counting label-expressing cells/5 sections because it does take into account if the section width is greater or smaller in one group versus the other. So if one genotype exhibited a wider length in the medial to lateral axis on a coronal section, the area and or cell number would be greater. However, by fixing cell counting to exactly 5 sections, a possible change in the anteroposterior axis is not considered. The authors should indicate that by assessing only 5 sections, they may not correctly estimate absolute cell number because changes in hippocampal size and extent may not be fully represented. They should also indicate that a more accurate approach would be to employ stereological methods. Nonetheless, the fact that cell numbers in the AhR-/- go from being in excess to no different with age suggests that the methods employed are indeed sensitive to major changes that occur with development. This concern applies to both Figure 2 and 5.

Rebuttal Comment 21). While the authors do address whether animals used in behavior were then used for IHC (the answer is no), and do describe the number of animal cohorts, and the order of testing, they fail to present this information in the Methods. Your readers will need to know this, please provide this information in the MS.

Rebuttal Comment 30). The discussion of using only a single foot shock for the AhR-icKO is very informative, and does indicate that the constitutive KO has features that differ from the C-KO. Please add the information presented in the rebuttal letter regarding using 1 vs 3 foot shock protocol into the MS, in the behavioral measures Methods and perhaps in the Results text, so the reader can appreciate these genotype distinctions. The authors might consider bringing up this issue in Discussion, but this is not required.
